# Data-Driven Platform Framework for Digital Whole-Process Expressway Construction Management

**DOI:** 10.3389/fnins.2022.891772

**Published:** 2022-06-02

**Authors:** Shu-Yang Chen, Jing-Xiao Zhang, Qi-Chang Ni, Martin Skitmore, Pablo Ballesteros-Pérez, Yong-Jian Ke, Jian Zuo, Hao-Jie Sun

**Affiliations:** ^1^School of Economics and Management, Chang’an University, Xi’an, China; ^2^Huaxi Company Build Installation Group Co., Ltd., Xi’an, China; ^3^Faculty of Society and Design, Bond University, Robina, QLD, Australia; ^4^Project Management, Innovation and Sustainability Research Centre (PRINS), Universitat Politècnica de València, Valencia, Spain; ^5^School of Built Environment, University of Technology Sydney, Sydney, NSW, Australia; ^6^School of Architecture and Built Environment, The University of Adelaide, Adelaide, SA, Australia

**Keywords:** data-driven platform, digitalization process, expressway, smart construction, BIM

## Abstract

To increase the speed and efficiency of expressways construction, information management is being gradually introduced into the construction process. However, progress is limited due to the complexity of expressway engineering and application limitations of information technology. Design and delivery are still dominated by paper files, and the management of test and inspection data is still relatively extensive. Research to date into digital expressway construction has been piecemeal and fragmented with a lack of research related to the whole construction process and a data-centric information management system yet to be realized. In response, through literature research and semi-structured interviews, the framework of a data-driven digital whole-process highway construction management platform was determined. A whole process management platform was established according to the framework, and the functional application of the proposed platform was explained through a case. The framework is proposed from the perspective of the whole process of collaborative sharing., which provides a new way of thinking to solve the problems existing in the current field of expressway construction whole-process management. It also provides data-centric management, electronic design and delivery, a refined workflow, and an efficient management process.

## Introduction

Expressways have developed rapidly to be one of the major types of infrastructure construction projects in China. The country’s total mileage of expressways reached 161,000 km in 2020, representing an annual increase of 7.62%. Expressway construction management is also gradually adopting digital methods, although the complexity and particularity of these projects itself present a certain degree of difficulty for digital management (Xie et al., 2011). From the perspective of engineering construction, the construction period of highway engineering is longer, the engineering construction environment is more complex, there are many factors influencing the construction process (Xiao et al., 2020), and projects need to be completed in a short time and with high quality. The requirements for construction quality and site safety are also very high.

From the perspective of information and data, highway engineering involves a large amount of data and a wide range of areas; data collection, analysis, and processing speed requirements are high; data have high commercial value; and data types are complex – including structured data and unstructured data, such as 3D drawing information, Building Information Modelling (BIM) dynamic display of information, and real-time construction control information. In addition to BIM, various digital technologies have been widely promoted and applied, with such new technologies as GIS, big data, and the Internet of Things helping in the development of digital engineering (Olawumi and Chan, 2019; Li et al., 2020; Zhang et al., 2020b; Prabhakaran et al., 2021).

However, due to their complex and special characteristics and the limitations of digital engineering technology, the current digital management of expressway construction has several shortcomings:

(1) Data-centric management has not been formed. At present, the digitalization of the engineering quality of various expressway construction projects mainly focuses on the preservation of traces of the quality management process, and most of the test/inspection data and production process data that play a decisive role in the quality of the engineering are only electronically archived in the test account (Yu et al., 2018; Xiao et al., 2020). In addition, the centralized storage of monitoring data of the Internet of Things has not yet formed a scientific management system with data management at the core (Kusano et al., 2011). This will lead to difficulty in information traceability, large barriers to information transmission between stages, and slow feedback on problems, resulting in low overall management efficiency (Xu et al., 2019).

(2) In terms of design, traditional paper delivery is still the norm (Kusano et al., 2011). There is little research into the digital delivery of design information. Road construction is less digitized, and there is a lack of research into information management for the integration of design and delivery information over the entire lifecycle of engineering projects (Zhang et al., 2020a). This results in many difficulties in establishing a highway BIM model, and in design information problems such as data loss, poor coordination and sharing.

(3) The management of test/inspection data is still relatively extensive. According to current construction, there is a large amount of test data such as construction self-inspections, supervision random inspections, intermediate delivery inspections, completion (delivery) acceptance inspections, and construction unit and industry management department law enforcement inspections (Zhang et al., 2019a). Although the data cover the entire process of highway construction, they are managed separately by each party and are yet to be linked together (Xiao et al., 2020; Pan et al., 2021). This makes the coordination of test and detection information rather poor and increases the difficulty of management.

A particular issue is that previous studies mainly focus on the necessity of expressway digital construction and the digitization of a single construction stage (Song et al., 2011; Xu et al., 2019; Zhang et al., 2020b). That is, there is no consideration for the digital construction technology of the whole process; whereas, a digital system for the whole construction management process has potential to provide a basis for collaborative work for all involved systems. It uses the same database to integrate project-related information for sharing, interoperability, and transmission; and the entire project measurement data, design data, construction data, and inspection data can be applied on the same platform. It can maximize the collaborative management of construction quality, and increase productivity, communication efficiency, and cost control. In addition, it breaks through the multiple separation aspects in the handover, change, and communication between design units, construction units, and supervision units in traditional project management – thereby greatly improving the construction management of engineering projects.

Therefore, this research needs to solve the following three problems: How to form a data-centric whole-process management system? How to integrate design delivery information during the design phase? How to realize the collaborative management of test data? This article introduces the entire process of digital management methods. Basically, through literature research and semi-structured interviews, we build a framework of a data-driven digital whole-process highway construction management platform. Then, the whole process management platform is constructed according to the framework, that is, the system can coordinate the measurement, design, construction, and inspection work in the process of expressway engineering construction. Compared with traditional project construction, the digital system for the whole construction management process based on BIM, provides a basis for collaborative work for all systems involved.

From a theoretical viewpoint, the framework is proposed from the perspective of the whole process of collaborative sharing. This provides a new mode of thinking for solving current problems in the management field of the whole process of highway construction. In addition, the specific process of highway digital construction technology based on collaborative theory is proposed, which is helpful to improve the information transmission dilemma of each construction stage of highway engineering for realizing the transmission and sharing of engineering information at each stage, thereby promoting the related research of highway digital process.

From a practical point of view, taking the Cambodian Phnom Penh-Sihanoukville project as an application example, the introduction of digital construction technology based on BIM will help companies upgrade their project management and control practices. In addition, the technology is based on a variety of advanced digital construction hardware and 3D model software. They enable collaborative control of project design, process, quality, production data, resource efficiency and interactive communication, helping to form a data-centric management system. In terms of design delivery, an electronic design information data storage and collaborative sharing mechanism can be formed. High-precision BeiDou positioning technology is used to accurately control real-time return of 3D design data, operation status and construction information. It also allows the digital control of process management, which can be changed at any time in case of design changes. Also, test and inspection data are interrelated and coordinated to ensure the accuracy of inspection items. In addition, the collected data can be used to predict the specific situation of the project, which can help personnel to make more objective decisions. Due to the electronic data storage, it also has a self-supervision effect on the implementation of enterprise projects, which has a positive significance for improving its management level.

The remainder of this article is organized as follows. Section 2 introduces BIM digital technology, digital construction paving, and some highway construction relevant facts. Section 3 introduces the basic theories involved. Section 4 describes the BIM-based construction technology framework for the whole process of highway digitalization. Section 5 describes the case study, Section 6 a discussion, and Section 7 concludes the paper.

## Literature Review

### Building Information Modeling Digital Technology

Building Information Modeling technology uses visual models as information carriers (Saka et al., 2020). BIM is frequently conceived as the digital expression of construction projects along with their geometric and non-geometric characteristics (Lu et al., 2017). It relates information of some elements with others, and it helps in the decision-making, implementation, and operation and maintenance by building a visual information systema and dataset. This has played a revolutionary role in the lifecycle management of many construction projects (Li et al., 2020; Zhang et al., 2020b; Banerjee and Nayaka, 2021).

An information management system, built on BIM technology, can store, calculate, and share large quantities of information from design, construction, and operation processes. This three-dimensional model can also help the management and efficiency of other non-construction tasks as well.

Compared to the building industry, BIM technology started late in the field of urban roads (Tang et al., 2020). However, it has developed rapidly in the past 2 years (Jin et al., 2017), with industry gradually paying more attention to this technology. Much experience has also been accumulated from real construction projects (Eadie et al., 2013; Li et al., 2018; Yang and Chou, 2018; Oraee et al., 2019). It has been proven in fact that applying BIM to basic road traffic construction can greatly help in construction organization and management. It can also improve the level and efficiency of information sharing at all construction stages, and promote the digital development of basic road transport (Li et al., 2020; Babatunde et al., 2021).

Also of relevance to the present study is that the application of BIM provides an opportunity for performing fundamental wider applications regarding both digitization and industrialization (Babatunde et al., 2019). Wan et al., for example, combined BIM with GIS to build a visual platform for bridge construction management (Wan et al., 2019). Zhu et al. combined BIM and 3D GIS to create a three-dimensional visual display and information management architectural BIM model for spatial analysis and measurement (Zhu and Wu, 2021). Kang and Hong designed a BIM/GIS-based software architecture for the effective integration of facility management data that laid the foundation for building a network service platform (Kang and Hong, 2015).

To date, the gradual integration of digital technology into infrastructure construction has already allowed increasing the speed of development of digital highway construction (Olawumi and Chan, 2019). For example, Song et al. analyzed the characteristics of expressway construction and explained the necessity for their digital construction (Song et al., 2011). Liu et al. developed automatic inspections that allowed video surveillance, visual analysis of spatial information, and video coding (Liu et al., 2021). Zhang et al. proposed the idea of digital expressway management (Zhang et al., 2020b); Xu et al. treated the application of digitization in highway management and maintenance (Xu et al., 2019); and Huang et al. introduced a tunnel engineering early warning control system to ensure construction safety and quality (Huang et al., 2017).

### Digital Construction Paving

Paving technology is one of the key elements in the process of digital construction of expressways. Its rapid development has helped improve the quality of high-grade pavements (Qin et al., 2018). This technology directly affects the flatness and compactness of the pavement (Deng et al., 2020), and thus affects the quality and service life of the whole infrastructure (Hu et al., 2017; Wan and Jia, 2019).

An asphalt concrete paver spreads the mixed asphalt concrete material evenly and densely on the pavement subbase or base layeIn the field of construction, because the construction period is generally long, with their. This is done with a certain degree of pre-compaction shaping to form the asphalt concrete base layer or surface layer (Huang et al., 2020; Hu et al., 2021). The use of asphalt concrete improves construction speed, save costs, and improve the quality of the road surface (Liu et al., 2018a; Nandi and Ransinchung, 2021; Zhu et al., 2021). Asphalt concrete also plays a key role in high-grade highways (Plati et al., 2016; Karpushko and Bartolomei, 2017). Nowadays, it is widely used in the paving operations of urban roads, large freight yards, parking lots, docks, and airports.

The quality of the work has a very important impact on the performance of the pavement. It is therefore necessary to control the quality of the paving and compaction processes to ensure the eventual quality of the pavement (Zhang et al., 2019b; Pradena et al., 2020). However, due to the usually complex conditions of construction sites, it is difficult for personnel to pave and roll the pavement in strict accordance with the technical requirements. On-site supervision and management personnel may also neglect irregular construction, resulting in inferior pavement quality (Makarov et al., 2021b). Therefore, with recent advances of technology, there is a general trend to apply digital technologies for monitoring and control purposes (Liu et al., 2016, 2020). This means that BIM-based highway construction technologies can also effectively improve the quality of paving and rolling, and improve the overall construction efficiency.

### The Whole Process of Expressway Construction

The importance of the whole process of expressway construction (i.e., measurement, design, construction, and inspection) has been widely recognized (Wong and Zhou, 2015; Edirisinghe et al., 2021). For the construction industry, this refers to the use of information technology, process integration, and manual operations in all processes of project planning, construction, and operation to complete the integrated management of construction project data (Zhuang et al., 2021). The advantages of introducing information management are obvious as the efficient management and interaction of all construction engineering information reduces the repetitive investment in each system of the construction project, avoids the waste of resources, and ultimately improves the overall efficiency of the construction project (Zhang et al., 2018; Sadrinooshabadi et al., 2021). The introduction of construction whole-process management can shorten construction time by nearly 5%, and reduce the time spent on data query by 1/2 and the time spent on information interaction between 1/3 and 3/5. In the end, information interaction and sharing are completed more efficiently, and construction costs can be greatly reduced (Mesaros et al., 2021).

However, after the introduction of information management, there may be problems such as an increase in the initial investment time cost (Costin et al., 2018), an increase in the cost of personnel training (Kaewunruen et al., 2020; Villena et al., 2020), and other limitations the management approach may not be suitable for Moreno Bazan et al. (2020). According to the theory of technology acceptance model (TAM), the enhancement of user experience (Panopoulou et al., 2021) and the support of government policy (Jing et al., 2018) will greatly improve the acceptance of technology. In the field of construction, because the construction period is generally long, with the increase of time, the personnel in the project have increased their experience in using new technologies, and their resistance to new things has decreased (Qi et al., 2021; Uusitalo and Lavikka, 2021). Moreover, the support of government policies reduces the financial pressure to a certain extent, and the advantages of introducing information management will gradually increase.

Current research mainly discusses the necessity of digital construction of expressways and the digitalization of single construction stages (Song et al., 2011; Xu et al., 2019; Zhang et al., 2020b). However, there is a lack of relevant research on the whole-process digital construction technology of expressways. Therefore, this paper introduces collaborative theory into the digital management of the entire process, develops a digital management system platform for the entire process based on BIM technology, and coordinates the measurement, design, construction and testing in expressway construction.

## Research Methods

To improve expressway management practice and promote the adoption of expressway digital whole-process management, semi-structured interviews and experimental research were adopted in this study.

### Semi-Structured Interviews

Qualitative methods are suited for problems that need to be explored and obtain a detailed understanding. Semi-structured interviews with relevant personnel were conducted to obtain an in-depth understanding of the current challenges of digital whole-process management and discuss potential solutions.

(1) Interview design stage

A total of 22 practitioners engaged in highway engineering construction were selected for the semi-structured interviews. To obtain valid and relevant information, the respondents were asked to have at least 5 years of experience in any unit of surveying, design, construction and testing in expressway engineering projects. Respondents were stratified randomly to ensure that their sample held different positions, such as managerial and non-managerial. The respondents’ background information is shown in Table 1.

**TABLE 1:** Background information of the respondents.

Construction activity	Role	Respondent number
Measurement	Manager	No. 3
	Non-manager	No. 6; No. 11
Design	Manager	No. 4; No. 14
	Non-manager	No. 5; No. 7; No. 23
Construction	Manager	No. 1; No. 10; No. 18; No. 21
	Non-manager	No. 2; No. 9; No. 15; No. 16
Detection	Manager	No. 8; No. 13
	Non-manager	No. 12; No. 17; No. 19; No. 20; No. 22

(2) Interview stage

During the interview, the interview questions were presented flexibly according to the interview outline, and the whole process was recorded. The audio recordings were transcribed, and the transcribed texts were analyzed using the “topic analysis method” with NVivo12 software. The questions used in the interview are shown in Table 2.

**TABLE 2:** Interview outline.

Blocks	Questions	Node coding in NVivo
Analysis of functional requirements	(1) What is the objective of digital expressway management?	Q1
	(2) Who are the main stakeholders involved in the digital process management?	Q2
	(3) What information should be collected in the process of digital whole-process management?	Q3
	(4) Which effective and convenient methods should be adopted for digital whole-process management?	Q4
	(5) Which stages should be included in the whole process management?	Q5
	(6) How can managers effectively manage expressway projects according to the management system?	Q6
Operation convenience requirements analysis	(1) What are the needs in terms of portability of system operation in the digital whole-process management?	Q7
	(2) What are the requirements for information transmission among different stakeholders?	Q8
	(3) What are the management requirements for project documents?	Q9

The following lists the interview process of four typical respondents.

Q1: “What is the objective of digital expressway management?”

Interviewee A: “Data in important stages of construction is isolated, and the biggest difficulty is data integration.”

Q2: “Who are the main stakeholders involved in the digital process management?”

Interviewee A: “The main stakeholders are the design unit, the construction unit and the testing unit.”

Q3: “What information should be collected in the process of digital whole-process management?”

Interviewee A: “Measurement information; design 2D drawings; paving process information (paving temperature, working position, paver position, etc.), compaction process information (compacter route, speed, compaction strength, etc.), vehicle information (driver information, loading time, loading location, transportation route, etc.); inspection data information (retaining samples, recording results, data and locations, etc.).”

Q4: “Which effective and convenient methods should be adopted for digital whole-process management?”

Interviewee A: “The method of encrypting control points in the measurement phase can save costs; the data visualization of the design based on BIM, combined with the intelligent compaction and paving technology in the construction phase, can effectively save costs; in addition, Intelligent management and control of vehicles can be used to view vehicle trajectories in real time. Digital facilities can also be introduced in data retention and monitoring in the laboratory.”

Q5: “Which stages should be included in the whole process management?”

Interviewee A: “For example: drawing design, fixed-point stakeout data; 3D modeling; paving; laboratory data should also be included.”

Q6: “How can managers effectively manage expressway projects according to the management system?”

Interviewee A: “The data is easy to find and can be changed in time; through the dynamic data of construction, it is possible to check whether various indicators are normal; in addition, the driving range of the vehicle can be set, and the alarm can be automatically alarmed when the range is exceeded. This kind of process management can also improve safety.”

Q7: “What are the needs in terms of portability of system operation in the digital whole-process management?”

Interviewee A: “Data at different stages are displayed on one platform, which can facilitate management; update data in real time; and change erroneous data in time.”

Q8: “What are the requirements for information transmission among different stakeholders?”

Interviewee A: “Information of different construction stages can be seen on the same platform; people with different roles have different permissions; when problems are found, they can be changed.”

Q9: “What are the management requirements for project documents?”

Interviewee A: “Measurement and design data are shared and all electronic.”

Q1: “What is the objective of digital expressway management?”

Interviewee B: “The goal is to carry out digital management of the whole process of construction with data as the core.”

Q2: “Who are the main stakeholders involved in the digital process management?”

Interviewee B: “Design unit, construction unit and testing unit.”

Q3: “What information should be collected in the process of digital whole-process management?”

Interviewee B: “Measurement information; design drawings; paving process information (paving temperature, work site, paver position, etc.), compaction process information (compacter route, speed, compaction strength, etc.), transporter vehicle information (driver information, loading time, loading location, transportation route, etc.); inspection data information (retaining samples, recording results, data and location, etc.).”

Q4: “Which effective and convenient methods should be adopted for digital whole-process management?”

Interviewee B: “The method of encrypting control points during measurement can save costs; based on BIM, the data visualization of design can be realized, and the introduction of digitization in the construction stage can effectively save costs; in addition, intelligent management and control of vehicles can be used to view vehicle trajectories in real time. Digital facilities can also be introduced for data retention and monitoring in the laboratory.”

Q5: “Which stages should be included in the whole process management?”

Interviewee B: “Fixed-point measurement and drawing design; road compaction; inspection report management.”

Q6: “How can managers effectively manage expressway projects according to the management system?”

Interviewee B: “If there is a problem, you can easily find it through the management system and make corresponding modifications, which can save a lot of time; the quality of the paving can be checked directly through the system; the detected data can be subjected to simple statistical analysis.”

Q7: “What are the needs in terms of portability of system operation in the digital whole-process management?”

Interviewee B: “Different people can easily see the data they need, and when problems are found, they can be changed.”

Q8: “What are the requirements for information transmission among different stakeholders?”

Interviewee B: “Data at all stages can be shared; managers have more authority than ordinary employees; erroneous data can be changed in time.”

Q9: “What are the management requirements for project documents?”

Interviewee B: “Data at all stages can be shared; all files can be stored.”

Q1: “What is the objective of digital expressway management?”

Interviewee C: “Now the most important thing in digital management is to integrate data and link the previously isolated data.”

Q2: “Who are the main stakeholders involved in the digital process management?”

Interviewee C: “Design unit, construction unit and testing unit.”

Q3: “What information should be collected in the process of digital whole-process management?”

Interviewee C: “Measurement information; design 2D drawings; paving process information, compaction process information, transporter vehicle information; detection data information. “

Q4: “Which effective and convenient methods should be adopted for digital whole-process management?”

Interviewee C: “The data visualization of design based on BIM, combined with the intelligent compaction and paving technology in the construction phase, can effectively save costs; digital facilities can also be introduced in the data retention and monitoring of the laboratory. “

Q5: “Which stages should be included in the whole process management?”

Interviewee C: “Measurement, design, management of transportation vehicles, management of test reports.”

Q6: “How can managers effectively manage expressway projects according to the management system?”

Interviewee C: “When problems are found, changes can be made; construction process data can be viewed and changed in real time, and streamlined management improves safety; data can be changed to reduce construction time.”

Q7: “What are the needs in terms of portability of system operation in the digital whole-process management?”

Interviewee C: “Data at various stages can be shared; viewing information is not limited by time; problems can be changed when problems are found.”

Q8: “What are the requirements for information transmission among different stakeholders?”

Interviewee C: “The data of each stage is displayed on the same platform; the personnel of the design unit only have the authority to change the data of the relevant stage; the data can be changed when problems are found.”

Q9: “What are the management requirements for project documents?”

Interviewee C: “It can be easily visualized after being digitized; data can be shared at different stages.”

Q1: “What is the objective of digital expressway management?”

Interviewee D: “The core is data management, such as measurement stage, design stage, construction stage, and inspection stage.”

Q2: “Who are the main stakeholders involved in the digital process management?”

Interviewee D: “Design unit, construction unit and testing unit.”

Q3: “What information should be collected in the process of digital whole-process management?”

Interviewee D: “Measurement information; design 2D drawings; paving process information (paving temperature, working position, paver position, etc.), compaction process information (compacter route, speed, compaction strength, etc.); transporter vehicle information (driver information, loading time, loading location, transportation route, etc.); inspection data information (retaining samples, recording results, data and location, etc.).”

Q4: “Which effective and convenient methods should be adopted for digital whole-process management?”

Interviewee D: “BIM technology can realize the visualization of design data, and the digital integration of the construction phase can effectively save costs; in addition, intelligent management and control of vehicles can be used to view vehicle trajectories in real time. Data retention and monitoring in the laboratory can also introduce digital facilities.”

Q5: “Which stages should be included in the whole process management?”

Interviewee D: “Fixed-point measurement, drawing design, paving; test data preservation.”

Q6: “How can managers effectively manage expressway projects according to the management system?”

Interviewee D: “If there is a problem, you can easily find it through the management system and make corresponding modifications; the quality of the paving can be directly checked through the system; the data can be changed to reduce the construction period.”

Q7: “What are the needs in terms of portability of system operation in the digital whole-process management?”

Interviewee D: “The data of each stage is displayed on the same platform; the data is easy to find; the problem can be changed.”

Q8: “What are the requirements for information transmission among different stakeholders?”

Interviewee D: “Data at different stages are displayed on one platform, which can facilitate management; the authority of managers should be different from those of non-managers; wrong data can be changed in time.”

Q9: “What are the management requirements for project documents?”

Interviewee D: “Data in the construction stage can be shared with design data; the data can be simply counted; the storage file capacity is large.”

### Experimental Research

**FIGURE 1:**
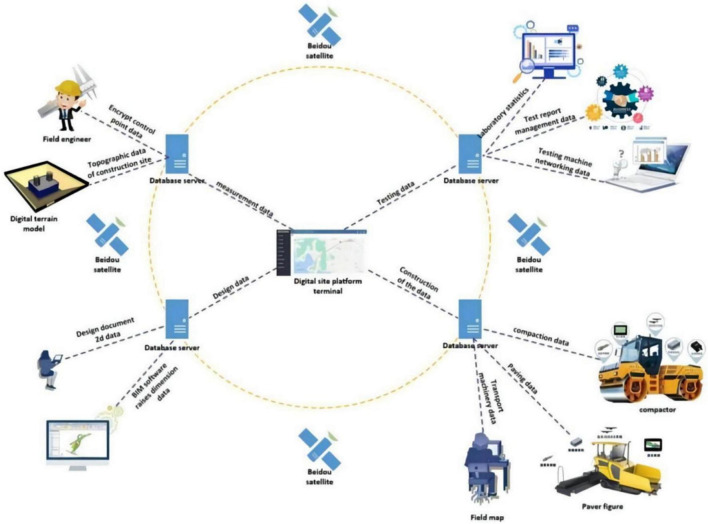
Topological diagram of the digital construction technology in the whole process of expressway construction based on BIM.

A system integrating BIM and BeiDou positioning technologies was designed as an experimental environment for verifying that information technology can address deficiencies in digital whole-process expressway construction management. To achieve this with multiple project participants, a BIM-based information platform was built with the collaboration of the authors and a Chinese company. The authors were responsible for requirements analysis, theory framework establishment, functional design, and 3D representation of the BIM model on a Web page. The Chinese company was responsible for system development including BIM model-loading technology, information transfer between different data formats, as well as the system prototype interface design. The system development for the experimental part of this research included six steps. Figure 1 shows a topological diagram of the digital construction technology in the whole process of expressway construction based on BIM.

Step 1: In the measurement phase, the use of BIM technology in digital whole-process construction allows the construction method to be simplified. The number of encrypted control points is reduced. The encrypted control point data information obtained from the measurement is uploaded to the digital platform terminal through the database server, and the surveying personnel use this digital platform. The terminal compares and checks the corresponding data and performs real-time updates. Based on control point measurement data and digital terrain analysis methods, the topographic morphology modeling and its attribute characteristics are expressed as numerical data. Through the realization of multi-level perception, multi-scale expression, and high-fidelity modeling of the terrain form, it provides a large amount of data information for the digital terrain of the project. These data are uploaded to the digital platform terminal through the database server and combined with the data information in the design stage. Thereby, an integrated three-dimensional spatial data model is defined, which can realize the coordinated management and seamless expression of the spatial data of the transit and its surrounding space.

Step 2: In the design stage, professional BIM software is used to transform the two-dimensional design drawings into a three-dimensional BIM model that can be recognized by the construction machinery. During the modeling process, the design drawings can be checked, design problems can be found through the BIM model, and design changes can be easily performed. The final BIM model data, combined with the digital terrain simulation data, can be uploaded to the digital platform terminal through the database server. This allows the integration of macro and micro information and, finally, the feedback to the digital system of each construction machinery. The latter prepares them for the construction operations with a one-time modeling. It also allows real-time linkage between different models. This is beneficial for the construction of multiple structural layers, which effectively improves design efficiency.

Step 3: In the construction phase, BeiDou navigation and positioning technology assist in the construction and management process of expressway engineering. Namely, it carries out “monitoring [tasks] during the event and retrospective [analyses] afterward” for the whole process of pavement construction. The system can transmit the key parameter indicators in road construction paving, compaction and transportation vehicle management. Then, it controls in real time the digital platform for statistical analysis of big data, thereby providing intelligent construction navigation in the whole construction site.

Step 4: In the inspection phase, the BeiDou high-precision GIS collection terminal is used to inspect and enter the construction quality of the project unit during construction. According to the quality specification, distinguishing the inspection requests and samples from the contracting unit and the supervision unit; recording the inspection time, data and location information; and uploading them to the platform in real time can ensure the accuracy and timeliness of the test data. This also ensures that the test results are traceable.

Step 5: Based on the results of the semi-structured interviews, a digital expressway construction management system is built to establish a comprehensive BIM-based framework for digital whole-process expressway construction. The system is capable of setting up different application modules in accordance with the digital expressway construction management process. It can also access the database and model information stored in the Cloud based on the technologies such as WebGL, dynamic model loading and BeiDou.

Step 6: Multiple stakeholders with different clearance access the test platform through the Web page. They use the digital whole-process expressway construction management framework to achieve a more meaningful collaborative management.

## Analysis of Interview Results

NVivo 12 qualitative data analysis software was used to conduct an in-depth analysis of the respondents’ data through topic analysis theory. Two respondents’ independently coded transcripts were used and we ensured discussions were held for every third transcript coded. After the information was saturated, we analyzed the transcripts one by one several times, and extract common themes through comparison. The results of the interview analysis are shown in Table 3. Four goals of the digital whole-process expressway construction management system were defined: (a) to allow data-centric management; (b) to accomplish electronic storage of design information and collaborative sharing; (c) to realize correlation and reconciliation of detection data, and (d) to improve the management efficiency of stakeholders.

**TABLE 3:** Grouping of identifiers characterizing the four thematic areas.

Thematic area	Identifiers
Data-centric whole-process management	Measurement stage; design phase; construction stage; detection stage; data-centric management
Electronic storage of design information and collaborative sharing mechanisms	2D drawing dimension upgrade; view and change within permissions
Correlation and collaboration of detection data	File sharing mechanism; inspection data information record
Improve the management efficiency of stakeholders	Visual management based on BIM; changeable data reduces wasted time; traceability issues; intelligent paving; intelligent compaction; intelligent management and control of transportation vehicles; intelligent detection; simple statistical analysis of data

### Enabling Data-Centric Whole-Process Management

Combined with the results of semi-structured interviews, the digital management of expressway mainly involved four stages: measurement stage, design stage, construction stage and inspection stage. It was necessary to implement data management at each stage. To allow this, synergy theory was used (Haken, 1984). Synergy theory can clarify the relationship and role of various elements/subsystems in the whole process of expressway construction, to promote the whole system from instability to stability and from disorder to order. Thereby, achieving the effect of 1 + 1 > 2. Precisely, it is through the coordination of information at different stages that more effective management of the whole process of expressway digitalization can be achieved.

### Electronic Storage of Design Information and Collaborative Sharing Mechanisms

During the design phase, the large number of images and calculations involved in traditional 2D drawings increases the probability of error. Using professional BIM software allows to build a 3D model. Upgrading construction drawings from 2D to 3D is the core of data management in the design phase. It handles all 2D data conversion, manages 3D design data, and handles all measurements, machine control tasks and analyses automatically. Correspondingly, on the basis of considering the results of semi-structured interviews, the design stage in the whole process of digital highway management should include functions such as upgrading 2D drawings, as well as viewing and changing relevant information under authorization.

### Realizing Correlation and Collaboration of Detection Data

According to current practices of expressway construction, there is a large amount of test data such as construction self-inspections, supervision random inspections, intermediate delivery inspections, completion (delivery) acceptance inspections, and law enforcement inspections. Although the data cover the entire process of highway construction, they are managed separately by each party and are yet to be linked together (Xie et al., 2011).

Combined with the results of semi-structured interviews, the management of the inspections stage in the process of highway digitalization management should mainly include two parts: the electronic record of inspection data and the file sharing mechanism.

(1) Electronic record of inspection data

The electronic record of inspection data refers to the register of the quality inspection information during the project construction stage. Based on the semi-structured interviews, the expressway whole process digital management system must be able to store and analyze various types of information related to the inspection processes.

(2) File sharing mechanism

Although the detection data covers almost the entire process of highway construction, they are managed individually by the parties and are not yet linked with each other. Based on the semi-structured interview results, in order to realize the digital management system of the whole process of expressway, our platform must be able to enable file sharing.

### Improving the Management Efficiency of Stakeholders

The information obtained or generated by the project personnel must be systemized. There is a need for an integrated system that can manage a series of tasks throughout the entire project. According to the semi-structured interview results, the following strategies to improve the efficiency of the whole process management of expressway digitalization are identified:

(1) Visual management based on BIM improves the coordination of all users and reduces communication barriers;

(2) Design data can be changed at any time when the design needs changes to reduce time waste;

(3) Problem can be traced back to the source, reducing the time cost of information retrieval;

(4) Intelligent management of paving, compaction and transportation vehicles, reduces labor costs and enables process-based operations;

(5) Simple statistical analysis of data helps to improve decision-making efficiency.

## Bim-Based Construction Technology Framework of the Whole-Process Expressway Digitalization

The technical framework of the research system platform is based on the sustainable infrastructure design framework established by Delft University of Technology’s scholars (Liu et al., 2018b), and incorporates the concept of whole-process collaboration; The scenarios when managers use the system and where it really works can be further understood through semi-structured interviews, so as to obtain the basic platform framework for the data-driven digital whole-process highway construction management.

**FIGURE 2:**
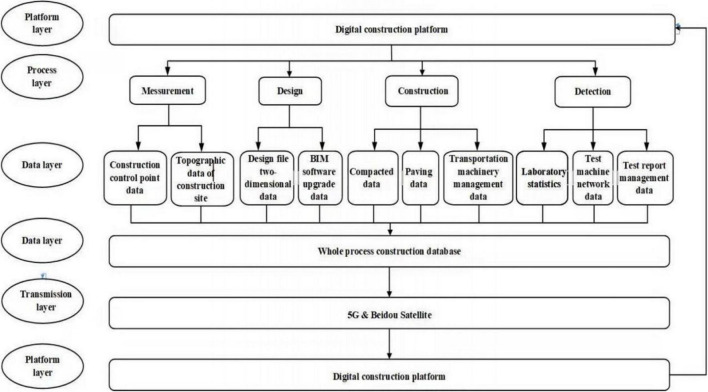
Technical architecture.

The highway digital full-process construction technology system is based on a variety of advanced technologies such as 3DGIS, BIM, and 5G networks. It combines measured data and two-dimensional design files to establish a highway BIM model and integrates and merges with the three-dimensional terrain to form three-dimensional visualization spatial environmental data of the highway and surrounding environment. Also, this system associates all kinds of data and information involved in the measurement, design, construction, and inspection stages with the model to form the comprehensive storage, management, and instant feedback of information. As Figure 2 shows, the technical architecture of the system platform includes four logical layers: the process layer, data layer, transport layer, and platform layer. Through four levels of collaborative work, the digitalized construction management of the expressway project can be finally obtained, the information transmission dilemma of each construction stage of the expressway project can be improved, and a new delivery method and a more efficient management system are provided for the expressway project.

After proposing the basic platform framework of data-driven digital whole-process highway construction management, the next step is to develop the corresponding whole-process system platform. The application of computer-aided software in construction can be traced back to 2009. Luong Duc Long et al. developed an expert consultation system about bridge structure and damage. The system can automatically detect the damage degree of bridge structure and strengthen emergency measures. The emergency measures for reinforcement treatment and the construction process of repair can be obtained after calculation (Long and Ohsato, 2009). In terms of construction site informatization management, Caroline P. Valente et al. proposed a set of design and evaluation methods to realize visual information management system according to the integration degree of construction site management process (Valente et al., 2019). However, the informatization of this platform only involves the construction stage, so this research, based on the synergy theory, integrates four management processes into the platform development.

### Encryption Control Point

Highway construction control includes a plane control network and elevation control network. The layout and measurement of its control points have corresponding principles and requirements. The use of BIM-based highway digital full-process construction technology is better than the traditional construction mode (a pair of reference piles staked out every 10 m), and only a high-precision robot-type total station is installed at 500 to 600 m intervals (in order to facilitate the installation of the instrument to adapt to complex working conditions, the instrument is generally set up in the rear rendezvous mode). The measured control point data information is uploaded to the digital platform terminal through the database server, and the surveyor compares and checks the corresponding data through the digital platform terminal and performs real-time updates and improvements.

### Digital Terrain Simulation

Based on the control point measurement data and digital terrain analysis methods, the topographic morphology modeling and its attribute characteristics are transformed into data. Through the realization of multi-level perception, multi-scale expression, and high-fidelity modeling of the topography, these topographic expressions provide a preliminary data basis for the external performance of the topography morphology. In addition, topographic factors are extracted, including slope, aspect, slope length, slope shape, slope position, and other slope topographic factors, as well as complex topographic factors, which reflect such comprehensive geographic features as the area elevation integral, topographic humidity index, and topographic dynamic index. Different terrain factors map different aspects of the landform and its process, and provide a large amount of data information for the digital terrain of analog engineering. These data information are uploaded to the digital platform terminal through the database server and combined with the data information in the design stage to form an integrated three-dimensional spatial data model. In this way, the coordinated management and seamless expression of rail transit and surrounding spatial data lay a data foundation for the design, construction, and inspection phases.

### Upgrading the Construction Drawings

In the design stage, the traditional paving technology calculates the points on the drawing to form a two-dimensional drawing based on the structures and structural layers designed on the control network and drawings. However, the large number of images and calculations involved may greatly increase the probability of error. The BIM-based highway digital full-process construction technology upgrades two-dimensional drawings with professional BIM software data to form corresponding three-dimensional BIM models before and after the upgrade, creating the electronic transformation of paper documents. The materials required for the dimension upgrade of construction drawings are a horizontal curve element table, vertical curve element table, and design every 20-meter cross-section diagram, plan diagram, pile-by-pile coordinate table, etc. Upgrading the construction drawings is the core of data management in the entire BIM-based highway digital construction technology process. It processes all two-dimensional data conversions, managing three-dimensional design data, processing measurement, mechanical control tasks, and analysis. Professional BIM software can intuitively import, review, and analyze graphical design information, and easily allocate, manage, and track construction information during the entire project.

The visualized 3D information of BIM technology is uploaded to the digital platform terminal through the database server, combined with the digital terrain simulation data information, to form an integrated 3D spatial data model. It can achieve effective collaboration among all parties involved and provide data during construction and inspection phases.

### Intelligent Control System of Construction Machinery

Professional BIM software upgrades the two-dimensional construction drawing to a three-dimensional BIM model that can be recognized by the construction machinery. Then, it checks the design drawings during the modeling process. Through the BIM model, finding design problems can adjust the design change model, and finally the BIM model can be distributed to the database in the digital system of each construction machinery item; the construction machinery is equipped with a digital full-process system based on BIM. In the construction process, the position and coordinates of the working parts can be obtained in real time and combined with the BIM model received by the digital construction control computer to realize the automatic construction operation of the working parts of the construction machinery based on the digital full-process system of BIM. Meanwhile, the system can upload the operation process data to the digital platform in real time, which can visually display the construction model and operation data. Finally, the system can use the detailed data returned by the digital whole-process system in real time to generate the BIM construction model and compare and analyze it with the BIM design model. The system can grasp the quality and progress of the construction operation in real time, make real-time adjustments in the subsequent construction process, and guide the subsequent construction.

#### Intelligent Paver Control System

**FIGURE 3:**
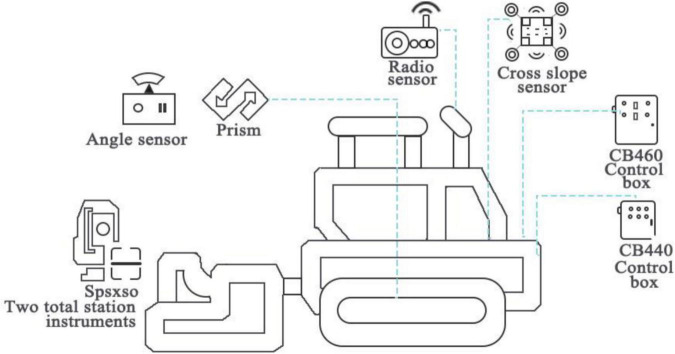
Topology diagram of intelligent control system of paver.

Before paving, the design of all pavers and handheld controllers should be checked, and the model re-checked. Using the BIM-3D driving view can help drive on a route, and visually display and check the precise surface terrain. During the construction process, the intelligent paving system of the paver uses the total station installed on the control point to transmit the captured MT900 coordinates to the CB460 control box of the paver control system through the data radio in real time. The control box is compared with the system-recognizable BIM model based on the design data, and then the elevation correction information is transmitted to the automatic control box CB440. Commands are issued by the control box, and the hydraulic cylinder is driven by the hydraulic valve to make a certain amount of displacement of the traction boom. The change of the position of the left and right traction points causes the vertical movement of the ironing board in the corresponding direction, so that the filling and building produce slope and elevation changes to compensate for road fluctuations. Therefore, it can achieve the required road surface smoothness and meet the design requirements. The real-time information collected by the equipment in the paving process, such as the location of the construction vehicle, driving speed, working time, and working location, is uploaded to the construction platform of the whole process, which can monitor and view the real-time operation information and guide the operation of the paving process. Figure 3 shows the topology diagram of the intelligent control system of the paver.

#### Intelligent Compactor Control System

**FIGURE 4:**
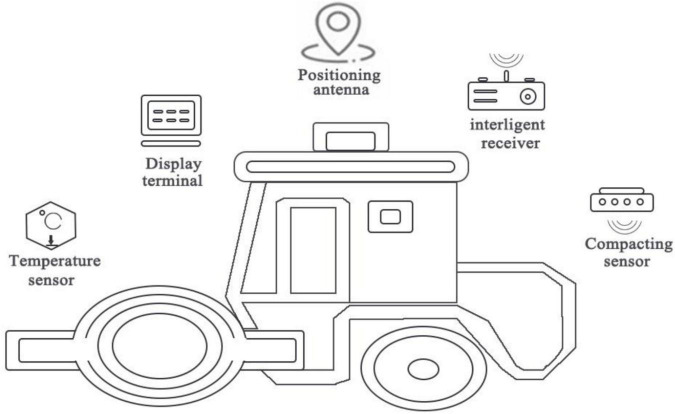
Topology diagram of compactor intelligent control system.

The intelligent compaction system uses the high-precision BeiDou + 5G positioning and compaction sensor technology to guide the roller’s rolling operations through the BIM model that can be recognized by the intelligent rolling system. The intelligent rolling system can display and record such physical parameters as the construction route, travel speed, compaction intensity, and vibration frequency required by the construction specifications digitally and graphically in real time, and guide the operator to real-time and effective construction to ensure the expected compaction indicators. The system transmits the compacted data to the digital platform terminal of the whole process in real time through the 5G gateway. Figure 4 is a topology diagram of compactor intelligent control system, showing that the BIM model is established through the collected real-time rolling data, and the rolling process is analyzed to guide the construction.

#### Intelligent Management and Control System for Transportation Vehicles

**FIGURE 5:**
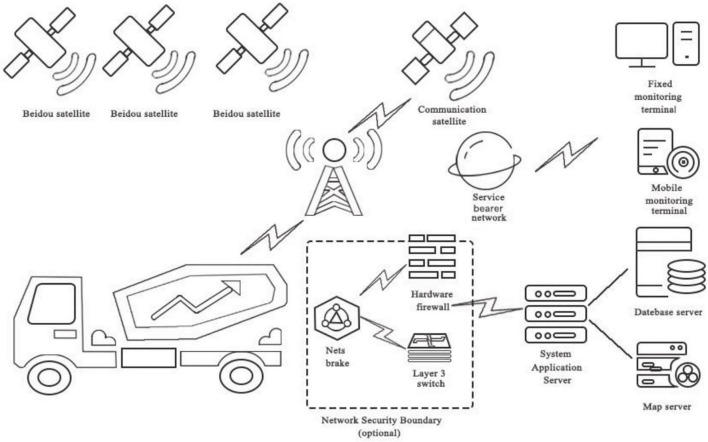
Topology diagram of the intelligent management and control system of transportation vehicles.

The intelligent management and control system of transportation vehicles uses radio frequency identification equipment and BeiDou positioning equipment to identify multiple information accurately, such as filler transportation vehicle information, driver information, loading time, loading location, transportation route, transportation time, and unloading warehouse surface. The system, reflecting the real-time location of the transport vehicle, has the functions of filler traceability and transport monitoring, which can connect the construction site and the stockyard, and ensure the transport vehicle arrives and unloads within the effective time. The location information can be transmitted back to the whole-process construction platform in real time *via* the network or self-organized WLAN covering the transportation distance. In addition, the intelligent management and control system for transportation vehicles can be equipped with electronic fences to manage and control transportation vehicles. Figure 5 shows the topology diagram.

### Intelligent Detection System

**FIGURE 6:**
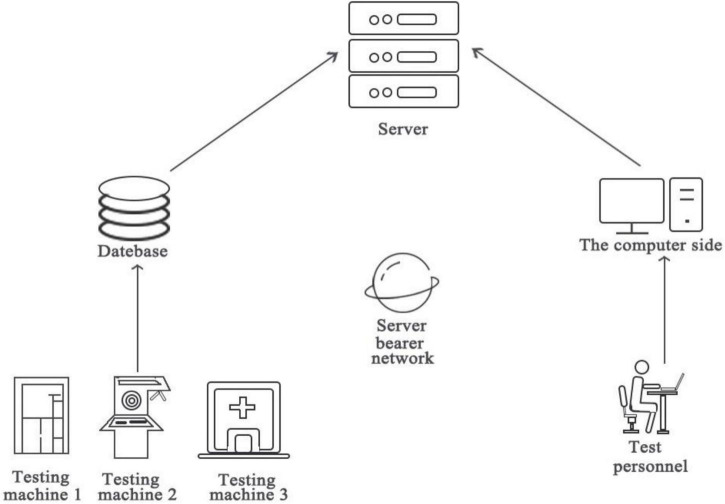
Topology diagram of intelligent detection system.

The detection system uses BeiDou high-precision GIS collection terminals and networked test machines to detect and input the unit project construction quality information in the process of project construction. According to quality management specifications, the inspection requests and reservations of contractors, supervision companies, and supervision organizations can be strictly distinguished, and the inspection results, data, and location are recorded separately. It can ensure the accuracy and precision of the inspection items and the authenticity and reliability of the test results. Figure 6 shows the topology diagram.

## Case Study

### Project Overview

**FIGURE 7:**
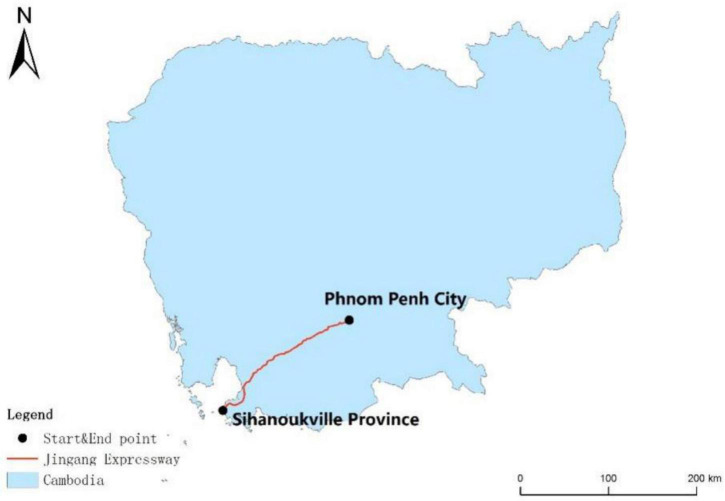
Geographical location of the Phnom Penh-Sihanoukville Expressway.

Once completed, the Phnom Penh-Sihanoukville Expressway will be the first expressway in Cambodian history. Opening up the capital economic circle and Cambodian largest deep-water port of Sihanoukville, it will have two-way four-lane roads and a design speed of 100 km/h. The total investment will exceed USD 2 billion. The construction period will be 4 years, and the operation period will be 50 years. Figure 7 shows its geographic location.

The total length will be 190 km, of which the Phnom Penh, Kandal province, Kampong Speu province, Koh Kong province, and Sihanoukville province sections will be 8.3 km, 9.1 km, 80.8 km, 1.92 km, and 89.89 km, respectively. The expressway will adopt the BOT model to invest and build. After completion, the journey from Phnom Penh to Sihanoukville Autonomous Port will be shortened by more than 3 h, which will greatly reduce logistics costs, and drive economic development and local employment along the route. It will also play a positive role in promoting the social and economic development of Cambodia.

Nizhandari, the director of the Cambodia 21st Century Maritime Silk Road Research Center, who has been tracking the “Belt and Road” project for a long time, has said that many industries in Cambodia have been severely affected by the COVID-19 epidemic, but the Phnom Penh-Sihanoukville Expressway project will serve as a “stabilizer” for Cambodia’s economic development. The Sihanoukville Port Special Zone had attracted 166 enterprises and created nearly 30,000 job opportunities. The construction of Cambodia’s first highway, the Phnom Penh-Sihanoukville, with an investment of more than USD 2 billion, is progressing smoothly. The current construction progress is more than 70% complete. In addition, the project has attracted much attention locally and internationally, which also endorses its significance (see https://www.crbc.com/site/crbc/295/info/2021/46884290.html).

The expressway project has five major characteristics: “high,” “new,” “difficult,” “risky,” and “tight.” As a key project of China-Cambodia cooperation under the framework of the “Belt and Road,” its quality requirements are high; the project adopts an innovative integrated management model, introduces the management mechanism of the consortium supervision unit, and incorporates the supervision and design units into the management team of the general manager department.

Factors such as the hot and wet climate of the geographical location, the remote transportation of materials, and the difficulty of communication with overseas engineers have increased the cost and difficulty of management and control. The geological conditions are complex and changeable, leading to high construction quality and safety control risks; and the high temperature and wet climate greatly reduce the actual effective working days. Based on these reasons, the project has introduced BIM and digital management technology, established a digital construction site platform, created a digital construction site, and comprehensively improved quality and efficiency.

The reasons for choosing the Phnom Penh-Sihanoukville Expressway as a case, first of all, from the perspective of the social significance of the project, its social significance is huge. It is the first expressway in Cambodia’s history, which will drive economic development and local employment along the route, and will play a positive role in promoting Cambodia’s social and economic development; secondly, from the perspective of cultural communication, this project is an overseas project, and there is a big communication barrier between the staff, the whole process platform framework can be used in the construction to solve this problem. Thirdly, in terms of project management mode, the project adopts an innovative integrated management mode, and introduces the management mechanism of the consortium supervision unit, incorporating supervision and design into the management team of the general manager department. The complex management model can greatly reduce the communication cost after adopting the full platform management, and the project parties can better conduct comprehensive management. Finally, after adopting the whole-process construction platform in this project, under the requirement of ensuring the high quality of the project, the problems of tight construction period, high risk and difficult construction have been largely solved.

### Engineering Construction Data System

The project is based on BIM and digital terrain simulation technology, combined with measurement and design data to form an integrated three-dimensional spatial data model. It integrates a digital management system, whose digital management platform is based on the BIM three-dimensional space model, and can realize the collaborative management and seamless expression of construction data during the entire process of expressway construction.

#### Intelligent Paver Control System

**FIGURE 8:**
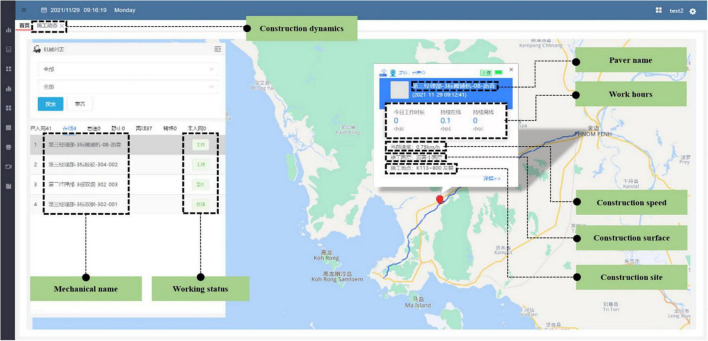
Digital platform paving information.

In the intelligent control system of the paver, the BeiDou positioning module and the equipment intelligent collector completes the data collection and transmission of construction parameters and transfers the real-time collected information of the construction vehicle position, driving speed, working time, and working position to the database for packaging to be sent. The construction data collected by the system can be sent to the digital information platform in real time through the 5G network, to complete the operation of receiving, displaying, and saving real-time paving information. Remote monitoring and management of the construction site can ensure the quality and efficiency of asphalt pavement construction. Figure 8 show the digital platform paving information.

#### Intelligent Compactor Control System

**FIGURE 9:**
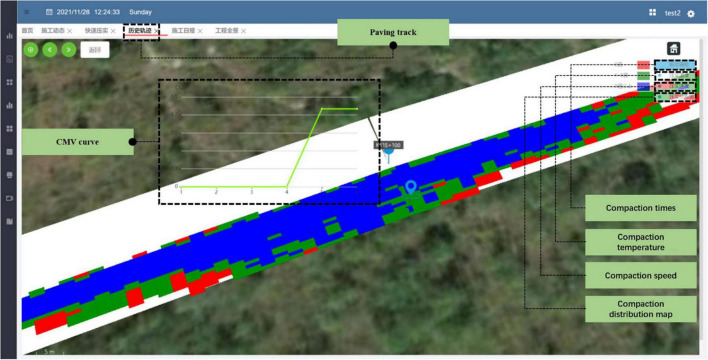
Intelligent control system of compactor.

The compactor intelligent control system can collect the parameters of the roller, such as rolling speed, compaction, amplitude, and other information, and use the BeiDou positioning module to obtain the latitude and longitude positioning. On the server, the roller compaction position can be obtained by latitude and longitude information, and the compaction process can be controlled. The parameter collection system of the roller can guide the amount of compaction through the detected compaction value, marking different colors in the road section information. According to the comparison between the set degree of compaction and the detected degree of compaction, the system can be combined with the degree of compaction result to guide the operator to roll. Figure 9 shows the intelligent control system of the compactor.

#### Intelligent Transportation Control System

**FIGURE 10:**
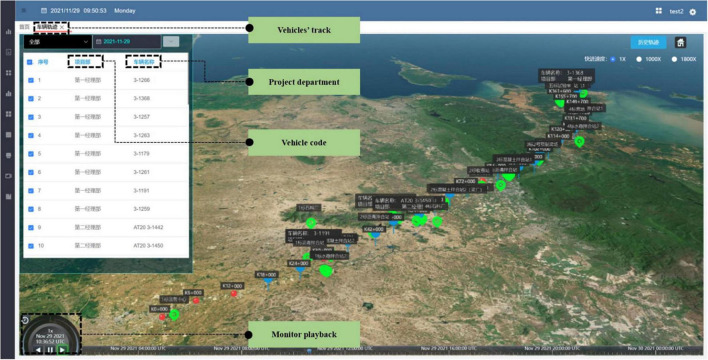
Vehicle trajectory.

As Figure 10 shows, in the digital construction platform, the intelligent transportation control of vehicles can be carried out, the information transmission interval can be independently set, and the trajectory of any truck in a certain period of time can be replayed on the whole-process construction platform. The system can also display the speed of the location. The fence area can be set on the electronic map. If the transport vehicle exceeds the fenced area, the system will automatically record and sound an alarm, which can accurately judge whether the trajectory of the transportation vehicle is normal or not.

### Engineering Inspection Data System

The engineering testing data system consists of three sections: laboratory statistics, test report management, and testing machine networking. The laboratory statistics section automatically analyses and calculates the original data, summarizing such data as the total number of test reports and total pass rate, total number of test machine samples and qualification rate, daily qualification rate of the test report, and daily number of samples of the testing machine. Information retrieval can also be carried out through the laboratory and time zone as required.

**FIGURE 11:**
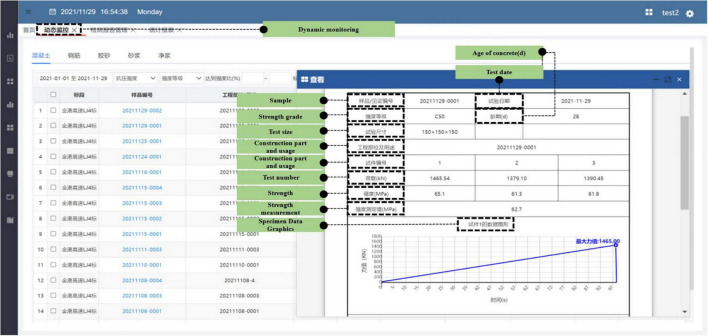
Test report management section.

In the inspection report management section, equipment data, sample ledger data, report ledger data, unqualified ledger data, statistical analysis data, supervision report ledger data, construction report ledger data, and supervision construction comparison ledger data can be managed. Taking testing equipment management as an example, the basic information data content of the management testing equipment includes the verification or calibration of the main equipment, equipment number, equipment name, specification model, manufacturer, testing department, equipment effective verification date, and affiliated inspection room. If the data lock status is unlocked, the manager can add, delete, or modify the device information. As Figure 11 shows, the equipment information can be retrieved by the subordinate bid section and equipment name or number.

The test machine networking section is divided into the dynamic monitoring of the experiment process and the statistical report. The dynamic monitoring can help trace such materials as concrete, steel bars, and mortar. For example, it can manage the bidding section of the concrete material, sample number, engineering location or application, strength grade, and even the magnitude of the force at different times. The components of the statistical report are the total number of tests, number of concrete tests, steel, cement tests, mortar tests, daily report of test results, statistics data of all test reports, and statistical data of unqualified testing machines. Those data can all be retrieved and exported by time period.

### System Application Results

The whole-process digital management system based on BIM technology is applied in the Phnom Penh-Sihanoukville Expressway project. The system associates various data involved in the measurement, design, construction and inspection stages with the model to form comprehensive storage, management and real-time feedback of information. The system provides different thinking modes for solving the five difficulties of the project.

(1) After the Phnom Penh-Sihanoukville Expressway project adopts the whole-process digital management system, the project quality has been greatly improved. The project quality has been greatly improved. And, some studies on construction quality evaluation had confirmed the idea that the introduction of digitization can improve the quality of engineering construction (Hu et al., 2017; Hosseini et al., 2020). The system promotes process operation mode and realizes standardized operation. Also, the system uses a comprehensive visual display interface to display the current construction status and real-time construction reports. And the construction effect can be controlled at any time on site, which can avoid possible rework caused by post-incident detection as much as possible. In addition, the system carries out data analysis and mining, decision support analysis in a targeted manner, and provides scientific decision-making basis for construction process management and control, thereby improving construction quality.

(2) The whole process digital management system has laid the foundation for the implementation of the innovative integrated management mode. The supervision and design units are included in the management team of the general manager department. Through the whole-process digital management platform, multi-party supervisors can view the real-time project at any time, and various departments at various stages of construction can provide timely feedback. In addition, through a comprehensive visual display interface, the barriers to professional requirements are reduced, and it is convenient for personnel in various departments to quickly understand the construction management situation.

(3) After adopting the whole-process digital management platform, the project difficulties such as the long-distance transportation of ground materials and the high cost of personnel communication in the Phnom Penh-Sihanoukville Expressway project have been largely solved. Regarding the vehicle management and control of the project’s ground material transportation, the system can provide active vehicle management and control measures according to the actual needs of command and dispatch management, and take measures such as drawing electronic fences, dynamic speed limits, and vehicle restrictions according to local conditions. Because the whole-process construction system cooperates with multiple stages of expressway construction information and displays it through comprehensive visual interfaces, it reduces the barriers to professional requirements and facilitates people of different cultures and languages to quickly understand the construction process.

(4) The Phnom Penh-Sihanoukville Expressway project has largely guaranteed the construction safety by applying the whole-process construction system. After adopting the system, on-site construction process operation mode was promoted to realize standardized operation. Standard construction work is easy for operators to control, reducing potential safety risk factors. Combined with the actual needs of command and dispatch management, the whole-process construction platform can draw measures such as electronic fences, main line dynamic speed limits, and vehicle restrictions according to local conditions, which greatly improves the safety of the construction site.

(5) The application of this system shortens the construction period of the Phnom Penh-Sihanoukville Expressway project. The use of the whole-process construction system can form a data-centric management system and improve the information transmission dilemma in each construction stage of the expressway project. This can reduce information transfer time. In terms of design delivery, changes can be made at any time in case of design changes. The system can work 24 h a day. The system uses the control box screen to display the current construction status, and the construction effect can be controlled at any time on site, avoiding possible rework caused by post-event inspection, and completing the project with the best quality in the shortest time. Time is an important factor in the evaluation of construction quality management (Ma et al., 2014; Abdel-Hamid and Abdelhaleem, 2022). And, in practice, under the premise of ensuring the construction quality, the contracting enterprise can complete the project in a short time. For the contract issuing unit, the quality of the construction project of the contracting enterprise is excellent. This means that while ensuring the quality of the project, the shorter the project time, the higher the construction quality.

## Discussion and Conclusion

### Discussion

This study adopted the whole-process digital management method, and developed the whole-process digital management system platform based on BIM technology, coordinating the measurement, design, construction, and inspection work in the process of expressway construction. The Phnom Penh-Sihanoukville Expressway project is used as an example application, introducing BIM-based digital construction technology to realize the whole process of digital construction of highway engineering. This not only provides a theoretical foundation for research into construction technology in the whole process of expressway digitization, but also promotes the actual process of expressway digitization.

From a theoretical viewpoint, the study is the first to incorporate synergy theory into the overall process management of expressway digitalization and analyze the feasibility of incorporating the synergy theory from the three major characteristics of highway digital management: complexity, openness, and non-linearity between subsystems. This provides a new mode of thinking for solving current problems in the management field of the whole process of highway construction. In addition, the specific process of highway digital construction technology based on collaborative theory is proposed, which is helpful to improve the information transmission dilemma of each construction stage of highway engineering for realizing the transmission and sharing of engineering information at each stage and promoting the related research of highway digital process.

From a practical point of view, digital full-process construction technology based on BIM technology provides a huge upgrade to project management and control, involving the digitization and intelligence of highway construction. This technology is based on a variety of advanced digital construction hardware and three-dimensional model software, and provides the coordinated control of project design, process, quality, production data, resource efficiency, and interactive communication, which helps form a data-centric management system. In terms of design delivery, it means an electronic design information data storage and collaborative sharing mechanism can be formed by adopting high-precision BeiDou positioning technology to accurately control the real-time return of 3D design data, operation status and construction information, and digital control of process management that can be changed at any time in the event of design changes. In addition, the test and inspection data are correlated and coordinated to ensure the accuracy of the inspection process, so the test results are true and reliable. The comparison of advantages and disadvantages before and after the introduction of digitization is shown in Table 4.

**TABLE 4:** Comparison of advantages and disadvantages before and after the introduction of digitization.

Construction type	Implementation of synergy theory	Implementation of information technology	Stages involved in digitization	Advantage	Disadvantage
Tradition	×	×	×	Good management adaptability; no development cost (Georgiadou, 2019)	High possibility of information transmission obstacles (Georgiadou, 2019); poor information traceability; unable to monitor site dynamics in real time (Costin et al., 2018), etc.
Digitization	×	√	Test stage	Scientific management can be achieved; cost savings (Georgiadou, 2019); security	There will be corresponding costs for both hardware and software (Georgiadou, 2019).
	×	√	Design stage	Easy data storage; reduction in information retrieval costs (Xiao et al., 2020)	Increased upfront time cost (Xiao et al., 2020; Zhang et al., 2020a)
	×	√	Construction stage	Improve efficiency while maintaining construction quality (Ding et al., 2019); environmental protection (Dong and Richards, 2018; Babatunde et al., 2019)	Poor management adaptability (Dong and Richards, 2018); there are information islands (Babatunde et al., 2019); high cost of personnel training
	√	√	Whole process	Data-centric management; time-saving when design changes; synergizing test data; high efficiency and ensuring construction quality; Cost saving and environmental protection	Increased upfront time cost; high cost of personnel training

In particular:

(1) A management system centered on data has been formed. This includes test and inspection data and production process data, which play a decisive role in the quality of the project, and a scientific management system with data management at the core. In terms of design delivery, the system forms an electronic design information data storage and collaborative sharing mechanism, adopts high-precision BeiDou positioning technology to accurately control the real-time 3D design data, operation status and construction information, and digitally control process management, which can be changed at any time in the event of design changes (Xiao et al., 2020); in addition, the test data can be correlated and coordinated to ensure the accuracy of the test items, and the test results can be true and reliable (Porter et al., 2014).

(2) It can improve efficiency and shorten construction period while ensuring construction quality. The system is not affected by light, and it can be constructed any time of day, even at night. Current construction status and real-time construction reports can be displayed by a control box screen to control the construction effect at any time on site, avoid possible rework caused by post-detection, and enable the project to be completed with the best quality in the shortest time (Ding et al., 2019).

(3) It can realize green environmental protection while saving costs. During the measurement process, there is no need for piling and lofting, which can reduce measurement costs (Babatunde et al., 2019; Makarov et al., 2021a). Also, the personnel experience requirements are low, and even inexperienced manipulators can achieve precise control. There is no need for many people, which can greatly reduce labor costs. In addition, it can improve the efficiency of machinery use (Georgiadou, 2019), and reduce the number and costs of rented machines and the consumption of oil and materials, while reducing the impact on the environment (Dong and Richards, 2018).

(4) It can be safer while standardizing operations. The specific application of the system can be in a process-oriented operation mode, realizing standardized operations, and easy for the operator to control according to the requirements of on-site construction technology so it can reduce potential safety risk factors (Jianchao et al., 2015).

### Conclusion

Based on BIM technology, this study adopts whole-process digital management methods to develop a whole-process digital management system platform and uses a variety of advanced digital construction hardware and three-dimensional model software to coordinate the measurement, design, construction, and inspection work in the process of highway engineering construction. It can coordinate and control project design, process, quality, production data, resource efficiency, etc., to solve the information transmission of each construction stage of a highway project. It can facilitate the transmission and sharing of engineering information at all stages. Hence, it can realize the transformation from managing “people” to “data,” to the transformation of production data from “people” to “machine and software,” and realize the transformation of management processes from people-driven to data-driven. In the end, the goal of reducing staff and increasing efficiency under the premise of ensuring quality can be achieved, and project management can be improved. This not only lays the foundation for future research into the complex construction process of expressway digitalization, but also promotes the process of expressway digitization. In addition, the collected data can not only be used for big data analysis, but also can be used to predict the specific situation of the project, which can help project personnel to make more objective decisions. Also, due to the electronic data storage, it has a self-supervision effect on the implementation of enterprise projects, which has a positive significance for improving its management level. The framework is proposed from the perspective of the whole process of collaborative sharing. The proposed framework provides further theoretical support for the secure information transaction field of blockchain technology.

In particular, further research is needed to address the study’s limitation of the system mainly feeding back the compaction situation of the entire road section through such information of the compaction equipment as the driving speed, trajectory, and compaction vibration; with the promotion and application of the system, it will continuously summarize and update road intelligent compaction technology (Kassem et al., 2015; Chen et al., 2021) and incorporate variables that affect compaction quality such as bituminous mixture into comprehensive consideration (Xu et al., 2012; Gong et al., 2021; Jiao et al., 2021), so that the real-time feedback of road compaction quality will be more accurate. It is also possible to further integrate the concept of coordinated construction of the fleet and develop the corresponding intelligent control software. With the further development of unmanned driving technology, the road compaction operation in the future will use unmanned on-site construction of the compaction equipment controlled by the full background. Finally, further research is needed to address to study’s limitation of covering just four (important) stages in the construction process by appropriately adding other stages to better match reality.

## Data Availability Statement

The raw data supporting the conclusions of this article will be made available by the authors, without undue reservation.

## Ethics Statement

Ethical review and approval was not required for the study on human participants in accordance with the local legislation and institutional requirements. The patients/participants provided their written informed consent to participate in this study.

## Author Contributions

S-YC and J-XZ: conceptualization. S-YC: methodology. J-XZ and Y-JK: Software. MS and H-JS: validation. PB-P, Y-JK, and JZ: formal analysis. JZ and Y-JK: investigation. J-XZ, PB-P, MS, and Q-CN: resources. S-YC, J-XZ, and Q-CN: writing – original draft preparation and review and editing. PB-P: visualization. J-XZ: supervision and project Administration. All authors have read and agreed to the published version of the manuscript.

## Conflict of Interest

Q-CN was employed by the Huaxi Company Build Installation Group Co., Ltd. The remaining authors declare that the research was conducted in the absence of any commercial or financial relationships that could be construed as a potential conflict of interest.

## Publisher’s Note

All claims expressed in this article are solely those of the authors and do not necessarily represent those of their affiliated organizations, or those of the publisher, the editors and the reviewers. Any product that may be evaluated in this article, or claim that may be made by its manufacturer, is not guaranteed or endorsed by the publisher.
